# Statement of Retraction: The myocardial infarction-associated transcript 2 inhibits lipid accumulation and promotes cholesterol efflux in oxidized low-density lipoprotein-induced THP-1-derived macrophages via inhibiting mitogen-activated protein kinase signaling and activating the nuclear factor erythroid-related factor 2 signaling pathway

**DOI:** 10.1080/21655979.2024.2299530

**Published:** 2024-12-09

We, the Editors and Publisher of the journal *Bioengineered*, have retracted the following article:

Fangxiang Mu, Yuqing Wang, Hong Wu, Qingxia You, and Daimin Zhng- 2022, The myocardial infarction-associated transcript 2 inhibits lipid accumulation and promotes cholesterol efflux in oxidized low-density lipoprotein-induced THP-1-derived macrophages via inhibiting mitogen-activated protein kinase signaling and activating the nuclear factor erythroid-related factor 2 signaling pathway, *Bioengineered* 13(1), doi: 10.1080/21655979.2021.2005932

Since publication, significant concerns have been raised about the integrity of the data and reported results in the article. When approached for an explanation, the authors did not provide their original data or any necessary supporting information. As verifying the validity of published work is core to the integrity of the scholarly record, we are therefore retracting the article. The corresponding author listed in this publication has been informed.

We have been informed in our decision-making by our editorial policies and the COPE guidelines.

The retracted article will remain online to maintain the scholarly record, but it will be digitally watermarked on each page as ‘Retracted’.

